# Unveiling the hidden link: fungi and HPV in cervical lesions

**DOI:** 10.3389/fmicb.2024.1400947

**Published:** 2024-08-26

**Authors:** Yulong Zhang, Lingsi Chen, Haibo Li, Yiling Zhuang, Qing Xie, Wenwen Li, Xia Yang, Xiangqin Zheng, Li Suyu, Huan Yi

**Affiliations:** ^1^Department of Gynecology, Fujian Province Key Clinical Specialty for Gynecology, National Key Gynecology Clinical Specialty Building Institution, Fujian Maternity and Child Health Hospital College of Clinical Medical for Obstetrics and Gynecology and Pediatrics, Fujian Medical University, Fuzhou, China; ^2^Fujian Maternity and Child Health Hospital, College of Clinical Medicine for Obstetrics and Gynecology and Pediatrics, Fujian Medical University, Fuzhou, China

**Keywords:** HPV, *Candida*, cervical lesions, p16, Ki67

## Abstract

**Background:**

Cervical cancer, primarily driven by high-risk human papillomavirus (HR-HPV) infection, ranks as the second most common cancer globally. Understanding combined infections’ role, including Cervical fungi, is crucial in cervical carcinogenesis. This study aims to explore the potential correlation between HR-HPV, cervical fungi, and cervical cancer, while adjusting for various factors.

**Methods:**

The study population comprised patients undergoing colposcopy and conization due to abnormal cervical screening results. Clinical data including age, gravidity, HPV (human papillomavirus) genotypes, cervical pathology, and p16/Ki67 expression were extracted. Cervical TCT (ThinPrep Pap Test) and HPV testing are utilized for screening cervical lesions, with fungal presence suggested by TCT results. 5,528 participants were included in this study. Statistical analyses investigated associations between HPV/fungi co-infection and cervical lesions, employing multinomial logistic regression and interaction analysis.

**Results:**

Co-infection with fungi and HPV may decrease the risk of cervical lesions compared to HPV infection alone. In the co-infection group, compared with HPV infection alone, the risk of low-grade squamous intraepithelial lesions (LSIL) was reduced by 27% (OR = 0.73, 95% CI: 0.59–0.90), the risk of high-grade squamous intraepithelial lesions (HSIL) was reduced by 35% (OR = 0.65, 95% CI: 0.51–0.82), and the risk of cervical cancer was reduced by 43% (OR = 0.57, 95% CI: 0.35–0.92). The interaction analysis revealed a negative interaction between fungal and HPV infections in the development of cervical cancer (RERI = −6.25, AP = −0.79, SI = 0.52), HSIL (RERI = −19.15, AP = −0.37, SI = 0.72) and LSIL (RERI = −1.87, AP = −0.33, SI = 0.71), suggesting a sub-additive effect, where the combined effect of the two infections was less than the sum of their individual effects. This indicates that fungal infection may attenuate the promoting effect of HPV on cervical lesions. In exploring the potential mechanism, we found that the co-infection group had significantly lower p16 positivity (54.6%) compared to the HPV-only group (60.2%) (*p* = 0.004), while there was no statistically significant difference in Ki67 positivity.

**Conclusion:**

This study unveils the intricate relationship between cervical fungi and HPV in cervical lesions. Co-infection with fungi and HPV against cervical lesions compared to HPV infection alone, indicating a novel clinical interaction. Lower p16 positivity in co-infection hints at a protective mechanism, urging further exploration.

## Introduction

Cervical cancer, ranking second globally among women’s malignancies after breast cancer (Thun et al., 2010), is primarily associated with persistent high-risk human papillomavirus (HR-HPV) infection, a prerequisite for cervical intraepithelial neoplasia (CIN) and cervical cancer (Wright et al., 2003). Although the HPV infection rate among Chinese women is 15.71%, and 84.6% experience HPV at least once, only a fraction progresses to cervical cancer (He et al., 2018). The majority of HPV and CIN I cases, and half of CIN II and 30% of CIN III cases, are self-contained (Richardson et al., 2003; Moscicki et al., 2010; Sun et al., 2011), and the immune system typically clears HPV infections within two years (Gravitt, 2011). Prolonged HPV presence, influenced by factors such as cervicitis or multiple sexual partners, results in a sustained high viral load (Malagutti et al., 2020), contributing to cervical lesions and their severity (Meijer and Walboomers, 2000; Snijders et al., 2003). Vaccines are an effective means of preventing HPV infection and reducing the risk of cervical lesions. Three commercial vaccines are available: bivalent Cervarix (HPV16 and 18), quadrivalent Gardasil (HPV6, 11, 16, and 18), and nine-valent Gardasil 9 (HPV6, 11, 16, 18, 31, 33, 45, 52, and 58). Despite the effectiveness of HPV vaccines, their adoption in developing countries remains slow (Kyrgiou et al., 2017). And obstacles surrounding HPV prevention and treatment of associated diseases are expected to remain in developing and economically disadvantaged areas (Chan et al., 2019). Thus, timely detection and intervention to block persistent HR-HPV infections remain a primary strategy for preventing cervical lesions.

Understanding and addressing changes in vaginal microecology and associated risk factors, as well as HPV16/18 viral load, is crucial for preventing and delaying the occurrence and progression of CIN/CC (Zhang et al., 2023). The maintenance of normal vaginal microecology, including anatomical structure, microecological flora, and local immunological and endocrine factors, is crucial (Mitra et al., 2016) in preventing genital tract infections. Alterations in the vaginal microecology have been linked with the development of cervical lesions (Liao, 2010). Vulvovaginal candidiasis (VVC) infections are common genital tract infections in women. Fungi observed in cervical cytology (TCT) may include *Candida* species, primarily *Candida albicans*, along with other fungi such as Aspergillus species. *Candida albicans* is an opportunistic pathogen that usually does not cause disease but may do so when immunity decreases or vaginal acidity changes (Donders et al., 2002). The increased inflammation, lactobacilli displacement, and the blocking of standard immune responses usually favor HPV infection development. These biofilm-forming pathogens can alter host immune responses, making it difficult for the immune system to eradicate these microbial complex communities. However, the impact of VVC on the natural history of HPV infection warrants further investigation. A meta-analysis found VVC to be a protective factor against HPV infection (OR 0.63, 95% CI 0.49–0.82, *P* < 0.05) (Liang et al., 2019), Additionally, a large cohort study revealed an inverse association between vulvovaginal candidiasis and cervical cancer risk (Jansåker et al., 2022).

Meanwhile, some studies concluded that *Candida* does not increase cervical cancer risk (Engberts et al., 2006). Moreover, *Candida* may enhance HPV vaccine immune response by stimulating T cell proliferation, suggesting a potential adjuvant role (Wang et al., 2013). However, few studies have assessed VVC and HPV/CIN relationships using cervical cytology results. Compared to vaginal fungal discharge and microbial culture tests, cervical cytology, observing cervical cell microorganisms directly under a microscope, offers superior assessment of Cervical Candidiasis. This method also offers better insights into the interplay between Cervical Candidiasis and HPV in cervical lesions. Further research on the links and mechanisms between Cervical Candidiasis and HPV/CIN could increase patient benefit.

With this study, our objective is to investigate the potential relationship between Cervical Candidiasis and cervical cancer, considering adjustments for age, lipid profiles, as well as parity, and other genital infections. P16 (INK4a) and Ki-67 can signify lesion/HPV load risks from HPV infection to some degree (Song et al., 2007). Additionally, we aim to concurrently explore their impact on P16 (INK4a) and Ki-67 expression. This comprehensive approach will provide insights into how Cervical Candidiasis and HPV may influence the molecular markers P16 (INK4a) and Ki-67 associated with cervical abnormalities.

## 2 Materials and methods

### 2.1 Study population

**FIGURE 1:**
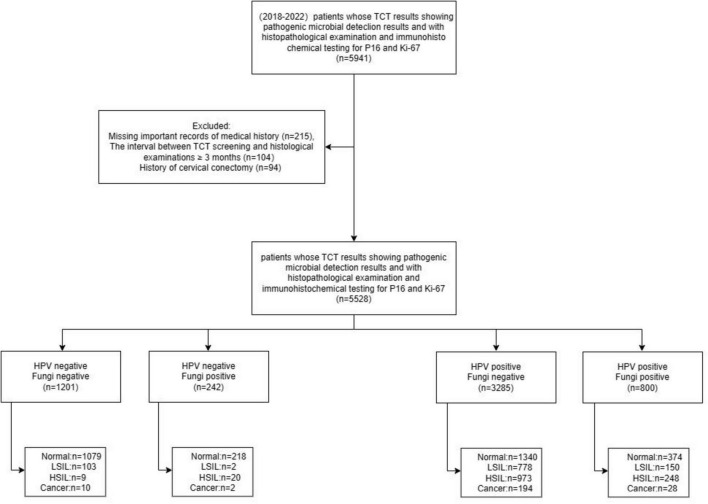
Flowchart of case inclusion process for analysis.

This cross-sectional study involved patients undergoing colposcopy and conization due to abnormal cervical cancer screening results at the Cervical Disease Center of Fujian Maternity and Child Health Hospital from June 2018 to December 2022. Cervical cancer screening included the ThinPrep Cytology Test (TCT) for both HPV and fungal detection, and/or HPV genotyping, with the interval between screening and histological examination being less than 3 months. Clinical information, including age, gravidity, parity, fungal infection status assessed by TCT, HPV genotypes, cervical pathology, and biomarkers such as p16 and Ki67 expression, was extracted from the department’s medical records. The study aimed to investigate the potential interaction between fungal and HPV infections in the development of cervical lesions, as well as to explore the underlying mechanisms, particularly the impact on HPV-related biomarkers. 5,528 participants were included in this study. The research design and case inclusion process are shown in Figure 1.

The study adhered to the Declaration of Helsinki (2013) and received approval from the Ethics Committee of Fujian Maternity and Child Health Hospital, Affiliated Hospital of Fujian Medical University (approval number: 2024KY032). Due to the retrospective nature of the study involving the analysis of existing medical records, the requirement for informed consent was waived by the Ethics Committee. However, all patient data were de-identified and handled confidentially to protect privacy.

### 2.2 Serologic detection and HPV genotyping

Cervical specimen collection and processing were performed using the ThinPrep Cytology Test (TCT) method, which allowed for the concurrent detection of HPV and cervical microorganisms. The presence of cervical microorganisms including trichomonas, bacteria, fungi, etc., detected in ThinPrep cytology test (TCT). The TCT samples were analyzed for the presence of *Candida albicans* and other fungal elements by experienced cytotechnologists and pathologists. HPV genotyping was performed using PCR-reverse dot blot (PCR-RDB) methodology (Yaneng Biotech) to identify high-risk HPV genotypes. This technique involves amplifying HPV DNA through PCR, then hybridizing the amplified products with type-specific probes on a membrane, allowing for simultaneous detection of multiple HPV types. The cytology and HPV testing processes followed standardized quality control protocols, and the laboratory personnel were blinded to the patients’ clinical information during the analysis. HPV genotyping was performed utilizing the PCR-RDB HPV genotyping method offered by Yaneng Biotech to differentiate among 18 types of high-risk HPV (HR-HPV) strains (16, 18, 31, 33, 35, 39, 45, 51, 52, 53, 56, 58, 59, 66, 68, 73, 82, and 83). Any identification of infection with a single high-risk HPV type was categorized as positive. Moreover, the presence of multiple HPV types in the sample was considered indicative of multiple infections.

### 2.3 Pathological diagnosis

Referrals for colposcopy followed the American Society for Colposcopy and Cervical Pathology (ASCCP) guidelines. All patients underwent colposcopy and cervical biopsy performed by qualified attending physicians at the Cervical Center of our hospital. Biopsy samples were obtained from abnormal areas using perforated cervical biopsy tissue forceps. The cervical biopsy specimens were then subjected to histopathological assessment in a blinded manner. Two senior pathologists independently examined the specimens and provided blind diagnoses regarding the presence of *Candida albicans* infection and other pathological findings. The histological endpoints were defined according to the World Health Organization (WHO) Classification of Female Genital Tumors (4th Edition) 2014, categorized as normal or cervicitis, low-grade squamous intraepithelial lesion (LSIL), high-grade squamous intraepithelial lesion (HSIL), and cervical cancer. Additionally, immunohistochemical staining was performed on the biopsy tissue specimens using a p16/Ki67 double staining kit, following the manufacturer’s instructions and technical guidelines for double immunostaining of cellular markers. The cervical specimens underwent processing in the histopathology laboratory, where a histopathologist blinded to the HPV status of the participants made the diagnosis. Immunocytochemical staining was carried out using the P16/Ki67 double staining kit on each cervical specimen. All experimental procedures strictly adhered to the kit instructions and technical guidelines for double staining of cervical cells. Two experienced pathologists performed and interpreted the double staining of cervical epithelial cells.

### 2.4 Statistical analysis

Participant characteristics were reported as mean ± standard deviation (SD) for continuous variables and as frequency and percentage for categorical variables. Comparative analysis of baseline characteristics across different cervical lesion categories was performed using one-way analysis of variance (ANOVA) or the Kruskal-Wallis rank-sum test for continuous variables and the Chi-squared test or Fisher’s exact test for categorical variables, as appropriate. Multinomial logistic regression models were employed to investigate the associations of separate and co-infection of HPV and *Candida albicans* with the risk of LSIL, HSIL, and cervical cancer. Multivariable-adjusted odds ratios (ORs) with corresponding 95% confidence intervals (CIs) were calculated, adjusting for potential confounders such as age, parity, and other relevant factors. Trend analysis was also conducted to assess the overall trend in the risk of cervical lesions across the different infection status categories.

Interaction analysis was performed to evaluate the potential interaction between HPV and *Candida albicans* infections in the development of cervical lesions. Measures of interaction, including the multiplicative scale, relative excess risk due to interaction (RERI), attributable proportion due to interaction (AP), and synergy index (SI), were calculated for LSIL, HSIL, and cervical cancer outcomes (Knol et al., 2011; Babatunde, 2021). Additionally, to elucidate whether the combined co-infection of HPV and *Candida albicans* influenced the risk of LSIL, HSIL, and cervical cancer through its impact on p16 expression, a biomarker of HPV-related oncogenic transformation, the positive rates of p16 expression were compared between different infection groups. Statistical significance was defined as two-sided, with a *p*-value ≤ 0.05 considered statistically significant. All statistical analyses were conducted using R software, version 4.2.2.^1^

## 3 Results

### 3.1 Basic characteristics and HPV-fungal co-infection analysis: variable summary

Table 1 presents baseline characteristics of participants. The study sample consisted of 5,528 participants, with 1,201 having neither fungi nor HPV, 242 with only fungi, 3,285 with only HPV, and 800 with both fungi and HPV. Statistically significant differences were observed across the groups for most variables. The mean age ranged from 35.7 years in the “Only Fungi” group to 40.3 years in the “Only HPV” group. The “Fungi & HPV” group had the lowest mean parity (1.3) and gravidity (2.1), while the “None” group had the highest (1.6 and 2.5, respectively), All *p*-values for these comparisons were less than 0.001. Regarding HPV classification, all participants in the “Only HPV” and “Fungi & HPV” groups were positive for HPV, while all in the “None” and “Only Fungi” groups were negative. The prevalence of trichomoniasis was low (< 1%) across all groups. The “Fungi & HPV” group had the highest prevalence of bacterial vaginosis (30.1%), followed by the “Only Fungi” group (12.8%). The “Only HPV” group had the highest proportion of high-grade squamous intraepithelial lesions (HSIL) (23.7%), while the “Fungi & HPV” group had the highest proportion of low-grade squamous intraepithelial lesions (LSIL) (31%). Blood lipid measurements, including A1c, total cholesterol, HDL-C, LDL-C, and triglycerides, showed some statistically significant differences across the groups, with the “Only Fungi” group having the highest mean A1c (1.4) and triglycerides (1.9 mmol/L).

**TABLE 1:** Basic characteristics and HPV-fungal co-infection analysis: variable summary.

Variables	Total (*n* = 5,528)	None (*n* = 1,201)	Only fungi (*n* = 242)	Only HPV (*n* = 3,285)	Fungi & HPV (*n* = 800)	*p*-value	Statistic
Age (mean ± SD)	39.7 ± 11.0	40.2 ± 10.6	35.7 ± 8.7	40.3 ± 11.3	37.3 ± 10.4	< 0.001	28.357
Parity (mean ± SD)	1.5 ± 1.3	1.6 ± 1.3	1.2 ± 1.2	1.6 ± 1.3	1.3 ± 1.2	< 0.001	12.025
Gravidity (mean ± SD)	2.4 ± 2.2	2.5 ± 2.2	2.1 ± 2.3	2.4 ± 2.2	2.1 ± 2.1	< 0.001	6.318
HPV classification (*n*%)						< 0.001	5,528
0	1,443 (26.1)	1,201 (100)	242 (100)	0 (0)	0 (0)		
1	4,085 (73.9)	0 (0)	0 (0)	3,285 (100)	800 (100)		
Trichomonas (*n*%)						0.004	Fisher
0	5,519 (99.8)	1,201 (100)	241 (99.6)	3,282 (99.9)	795 (99.4)		
1	9 (0.2)	0 (0)	1 (0.4)	3 (0.1)	5 (0.6)		
Bacteria (*n*%)						< 0.001	226.717
0	4,740 (85.7)	1,123 (93.5)	211 (87.2)	2,847 (86.7)	559 (69.9)		
1	788 (14.3)	78 (6.5)	31 (12.8)	438 (13.3)	241 (30.1)		
Cervical Classification (*n*%)						< 0.001	1,028.528
0	3,011 (54.5)	1,079 (89.8)	218 (90.1)	1,340 (40.8)	374 (46.8)		
HSIL	939 (17.0)	9 (0.7)	2 (0.8)	778 (23.7)	150 (18.8)		
LSIL	1,344 (24.3)	103 (8.6)	20 (8.3)	973 (29.6)	248 (31)		
Cancer	234 (4.2)	10 (0.8)	2 (0.8)	194 (5.9)	28 (3.5)		
A1.g.L. (mean ± SD)	1.3 ± 0.3	1.3 ± 0.3	1.4 ± 0.3	1.3 ± 0.3	1.3 ± 0.3	< 0.001	6.721
B.g.L. (mean ± SD)	0.9 ± 0.3	0.9 ± 0.2	0.9 ± 0.3	0.9 ± 0.3	0.9 ± 0.2	0.097	2.108
Total cholesterol (mean ± SD)	5.1 ± 1.2	5.1 ± 1.1	5.2 ± 1.3	5.1 ± 1.2	5.1 ± 1.1	0.019	3.334
HDL cholesterol (mean ± SD)	1.5 ± 0.4	1.5 ± 0.4	1.6 ± 0.4	1.5 ± 0.4	1.5 ± 0.4	0.017	3.382
LDL cholesterol (mean ± SD)	2.8 ± 0.8	2.8 ± 0.8	2.8 ± 0.9	2.8 ± 0.8	2.8 ± 0.8	0.101	2.08
Triglycerides (mean ± SD)	1.7 ± 1.3	1.6 ± 1.2	1.9 ± 1.5	1.7 ± 1.3	1.6 ± 1.2	0.002	5.091

ApoA1, apolipoprotein A1; ApoB, apolipoprotein B; TC, total cholesterol; TG, triglyceride; HPV, human papillomavirus; Fungi, fungi virus.

### 3.2 Association between HPV, fungi, and cervical lesions: odds ratios and 95% confidence intervals

Table 2, adjusted for age, pregnancy, parity, apolipoprotein A1, apolipoprotein B, total cholesterol, and triglyceride, presents the association between HPV, Fungi, and cervical lesions. Odds ratios (OR) and 95% confidence intervals (CI) are provided for LSIL, HSIL, and cervical cancer. The analysis includes participants grouped by HPV status, Fungi status, and their co-infection status. Among the total 5,528 participants, HPV infection was associated with significantly increased odds of LSIL (OR = 7.45, 95% CI: 5.93–9.37), HSIL (OR = 82.36, 95% CI: 38.91–174.3), and cervical cancer (OR = 12.07, 95% CI: 6.65–21.89) compared to no HPV infection. Fungal infection alone was associated with decreased odds of LSIL (OR = 0.75, 95% CI: 0.61–0.92), HSIL (OR = 0.65, 95% CI: 0.51–0.82), and cervical cancer (OR = 0.61, 95% CI: 0.39–0.97) compared to no fungal infection. Compared to individuals with neither HPV nor fungal infection, those with only HPV infection had significantly increased odds of LSIL, HSIL, and cervical cancer.

**TABLE 2:** Association between HPV, fungi, and cervical lesions: odds ratios and 95% confidence intervals.

Variable	Total	None	LSIL	HSIL	Cervical cancer	LSIL		HSIL		Cervical cancer	
						OR (95% CI)	*P*	OR (95% CI)	*P*	OR (95% CI)	*P*
**HPV**											
No	1,443	1,297 (89.9)	123 (8.5)	11 (0.8)	12 (0.8)	1(Ref)		1(Ref)		1(Ref)	
Yes	4,085	1,714 (42)	1,221 (29.9)	928 (22.7)	222 (5.4)	7.45 (5.93∼ 9.37)	< 0.001	82.36 (38.91∼174.3)	< 0.001	12.07 (6.65∼ 21.89)	< 0.001
**Fungi**											
No	4,486	2,419 (53.9)	1,076 (24)	787 (17.5)	204 (4.5)	1(Ref)		1(Ref)		1(Ref)	
Yes	1,042	592 (56.8)	268 (25.7)	152 (14.6)	30 (2.9)	0.75 (0.61∼0.92)	0.005	0.65 (0.51∼0.82)	< 0.001	0.61 (0.39∼0.97)	0.037
**HPV & Fungi**											
None	1,201	1,079 (89.8)	103 (8.6)	9 (0.7)	10 (0.8)	1(Ref)		1(Ref)		1(Ref)	
Only HPV infection	3,285	1,340 (40.8)	973 (29.6)	778 (23.7)	194 (5.9)	7.6 (5.93∼9.74)	< 0.001	73.68 (34.76∼156.16)	< 0.001	13.57 (7.14∼25.8)	< 0.001
Only fungi infection	242	218 (90.1)	20 (8.3)	2 (0.8)	2 (0.8)	0.84 (0.46∼1.52)	0.568	1.22 (0.36∼4.2)	0.75	1.55 (0.34∼7.09)	0.575
Fungi & HPV co-infection	800	374 (46.8)	248 (31)	150 (18.8)	28 (3.5)	5.61 (4.16∼7.57)	< 0.001	47.88 (22.04∼104.02)	< 0.001	7.8 (3.63∼16.74)	< 0.001
*P* for trend	5,528	3,011 (54.5)	939 (17)	1,344 (24.3)	234 (4.2)	1 (0.98∼1.02)	0.94	1 (0.99∼1.02)	0.702	1 (0.95∼1.04)	0.836

HPV, human papillomavirus; Fungi, fungi virus. Model adjusted for age, pregnancy, parity, apolipoprotein A1, apolipoprotein B, total cholesterol, triglyceride.

Compared to individuals with only HPV infection, those with co-infection of HPV and fungal infections had lower odds of developing cervical lesions. Specifically, the odds ratios for co-infection were lower than those for only HPV infection when considering the risk of low-grade squamous intraepithelial lesions (LSIL) (OR 5.61 vs. 7.6), high-grade squamous intraepithelial lesions (HSIL) (OR 47.88 vs. 73.68), and cervical cancer (OR 7.8 vs. 13.57). This suggests that co-infection with HPV and fungi may be associated with a reduced risk of cervical lesions compared to HPV infection alone, although both conditions increased the risk relative to no infection.

### 3.3 Subgroup analysis of HPV and fungi co-infection on cervical lesions

Table 3 illustrates a subgroup analysis examining the influence of HPV and Fungi co-infection on cervical lesions. Participants are stratified based on their HPV and Fungi status. Among the 1,201 participants with neither HPV nor fungal infection, the prevalence of LSIL, HSIL, and cervical cancer was 8.6, 0.7, and 0.8%, respectively. This group served as the reference category for comparison. In the subgroup with only fungal infection (*n* = 242), the odds ratios for LSIL, HSIL, and cervical cancer were not significantly different from the reference group (OR = 0.94, 95% CI: 0.51–1.74; OR = 1.12, 95% CI: 0.23–5.49; OR = 1.45, 95% CI: 0.26–7.96, respectively).

**TABLE 3:** Subgroup analysis of the association between fungal infection and cervical disease related HPV.

Subgroup		Total	None	LSIL	HSIL	Cervical cancer	LSIL	HSIL	Cervical cancer	*P* for interaction
							OR (95% CI)	OR (95% CI)	OR (95% CI)	
HPV	Fungi									
No	No	1,201.0	1,079 (89.8)	103 (8.6)	9 (0.7)	10 (0.8)	1(Ref)	1(Ref)	1(Ref)	
No	Yes	242.0	218 (90.1)	20 (8.3)	2 (0.8)	2 (0.8)	0.94 (0.51∼1.74)	1.12 (0.23∼5.49)	1.45 (0.26∼7.96)	0.401
Yes	No	3,285.0	1,340 (40.8)	973 (29.6)	778 (23.7)	194 (5.9)	1(Ref)	1(Ref)	1(Ref)	
Yes	Yes	800.0	374 (46.8)	248 (31)	150 (18.8)	28 (3.5)	0.73 (0.59∼0.9)	0.65 (0.51∼0.82)	0.57 (0.35∼0.92)	< 0.001

HPV, human papillomavirus; Fungi, fungi virus. Model adjusted for age, pregnancy, parity, apolipoprotein A1, apolipoprotein B, total cholesterol, triglyceride.

In the subgroup with only HPV infection (*n* = 3,285), the prevalence of LSIL, HSIL, and cervical cancer was 29.6, 23.7, and 5.9%, respectively. This group served as the reference category for comparison. Among participants with co-infection of HPV and fungi (*n* = 800), the odds of LSIL (OR = 0.73, 95% CI: 0.59–0.9), HSIL (OR = 0.65, 95% CI: 0.51–0.82), and cervical cancer (OR = 0.57, 95% CI: 0.35–0.92) were significantly lower compared to the subgroup with only HPV infection.

### 3.4 Interaction effects of HPV and fungi co-infections on cervical lesions after multivariable adjustment

**FIGURE 2:**
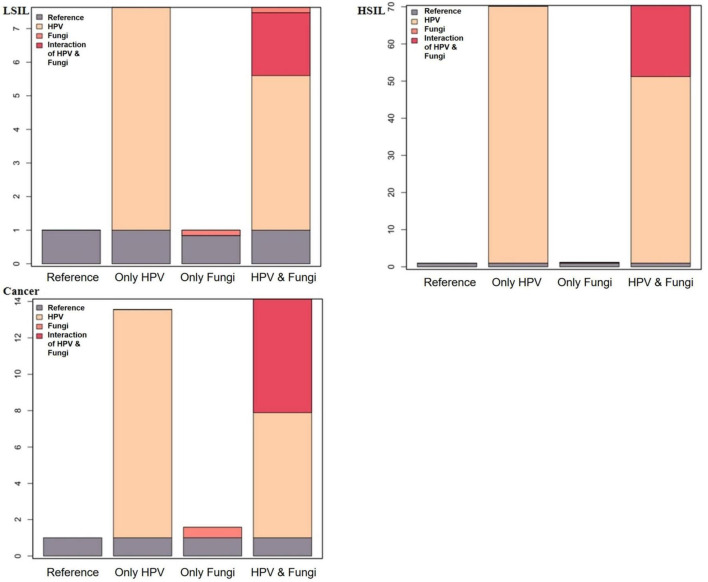
Additive interaction effect of fungi and HPV infections with LSIL, HSIL, and cervical cancer.

Table 4 and Figure 2 depict the interaction effects of HPV and Fungi co-infections on low-grade squamous intraepithelial lesion (LSIL), high-grade squamous intraepithelial lesion (HSIL), and cervical cancer after rigorous multivariable adjustment. The results indicate an antagonistic interaction between HPV and Fungi co-infections in the development of cervical cancer. The table presents measures of interaction between HPV and Fungi co-infection on cervical lesions, adjusted for age, pregnancy, parity, apolipoprotein A1, apolipoprotein B, total cholesterol, and triglyceride. For cervical cancer, the multiplicative scale indicates a 0.37-fold decrease in risk, but the confidence interval was wide, with RERI at −6.25, AP at −0.79, and SI at 0.52. The RERI (−6.25, 95% CI: −12.92 to 0.42) and AP (−0.79, 95% CI: −1.72 to 0.13) indicated a potential negative interaction. For HSIL, there’s a 0.6-fold decrease in risk, the confidence interval was wide, RERI at −19.15, AP at −0.37, and SI at 0.72. The RERI (−19.15, 95% CI: −36.83 to −1.47), AP (−0.37, 95% CI: −0.67 to −0.08) consistently indicated a negative interaction. For LSIL, the multiplicative scale is 0.88, RERI at −1.87, AP at −0.33, and SI at 0.71, the RERI (−1.87, 95% CI: −3.39 to −0.36), AP (−0.33, 95% CI: −0.64 to −0.03) all pointed toward a negative interaction. These findings suggest a negative interaction, indicating that the combined effect of HPV and Fungi co-infection is less than the sum of their individual effects on cervical lesion development.

**TABLE 4:** Interaction effects of HPV and fungi co-infections on cervical lesions after multivariable adjustment.

Measures	Estimates	95% CI low	95% CI up
**Cervical cancer**			
Multiplicative scale	0.37	0.07	1.86
RERI	−6.25	−12.92	0.42
AP	−0.79	−1.72	0.13
SI	0.52	0.29	0.94
**HSIL**			
Multiplicative scale	0.6	0.13	2.84
RERI	−19.15	−36.83	−1.47
AP	−0.37	−0.67	−0.08
SI	0.72	0.58	0.9
**LSIL**			
Multiplicative scale	0.88	0.47	1.65
RERI	−1.87	−3.39	−0.36
AP	−0.33	−0.64	−0.03
SI	0.71	0.54	0.93

HPV, human papillomavirus; Fungi, fungi virus; RERI, relative excess risk due to interaction; AP, attribute proportion; SI, synergy index. Model adjusted for age, pregnancy, parity, apolipoprotein A1, apolipoprotein B, total cholesterol, triglyceride.

### 3.5 Distribution of Ki67 and P16 expression by HPV and fungi co-infections

**FIGURE 3:**
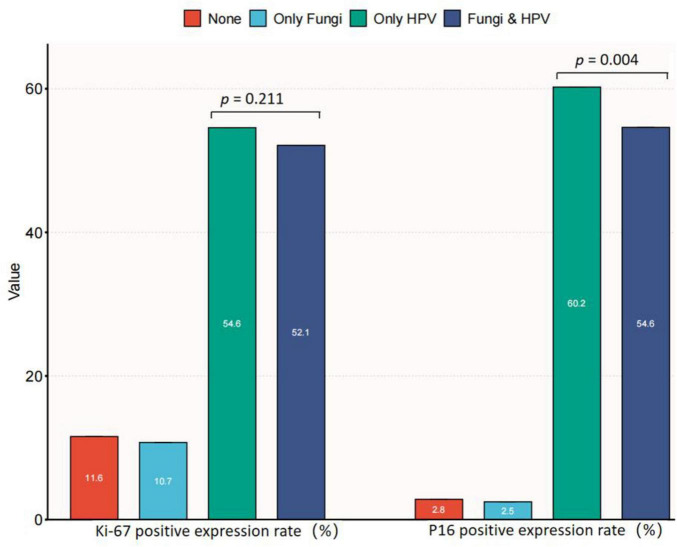
Distribution of Ki67 and P16 expression by HPV and fungi co-infections.

P16 (INK4a) and Ki-67 expression serve as indicators of lesion/HPV load risks and are associated with cervical abnormalities. The distribution of Ki67 and P16 expression in relation to HPV and fungal co-infections was analyzed. This analysis provides insights into how cervical candidiasis and HPV may influence these molecular markers. Figure 3 presents data on Ki67 and p16 expression in relation to the presence or absence of fungal and HPV infections among a total of 5,528 participants. The participants were categorized into four groups: Normal (*n* = 1,201), Only Fungi (*n* = 242), Only HPV (*n* = 3,285), and Fungi & HPV (*n* = 800). Regarding Ki67 expression, there was no significant difference between the two groups (*p* = 0.211), with 47.9% in the “Fungi & HPV” group and 45.4% in the “Only HPV” group showing no Ki67 expression. However, for p16 expression, a significant difference was observed (*p* = 0.004). A higher proportion of participants in the “Only HPV” group (60.2%) expressed p16 compared to the “Fungi & HPV” group (54.6%). The findings suggest that fungal co-infection with HPV may be associated with a lower risk of cervical lesions, potentially related to its impact on p16 expression. Among HPV-positive individuals, those with fungal co-infection had significantly lower p16 expression compared to those with HPV infection alone. However, no significant difference was observed in Ki67 expression between the two groups. These results indicate that the potential protective effect of fungal co-infection against cervical lesions could be linked to its influence on p16, a marker of HPV-related oncogenic transformation. The relationship between fungal co-infection and Ki67, a marker of cell proliferation, warrants further investigation to elucidate the underlying mechanisms.

## 4 Discussion

According to “Global Cancer Statistics 2018,” cervical cancer ranks fourth globally in female tumor incidence and mortality, and second in developing countries (Bray et al., 2018). While HPV infection is necessary but insufficient for cervical cancer (de Sanjosé et al., 2007), fewer cervical cancer cases are reported than HPV-infected females (Monnier-Benoit et al., 2006). Recent microbiome research has revealed a close link between changes in the cervical-vaginal microenvironment and HPV-related cervical cancer (Mitra et al., 2016; Huang et al., 2018). A comprehensive analysis of 24 studies (Zhang and Begg, 1994), primarily comprising case reports or case series, showed a notable correlation, further supported by a 2018 meta-analysis of 17 observational studies (Yang et al., 2018), indicating a positive association between prolonged television (TV) exposure and the risk of cervical cancer development. However, the correlation between *Candida* infection and HPV remains uncertain, and its impact on precancerous cervical lesions is also undetermined. Studying the potential link between *Candida* infection and HPV is crucial for understanding their correlation and impact on precancerous cervical lesions. This research may provide insights for developing effective strategies in cervical cancer prevention and treatment, offering a new perspective for early screening and intervention. Confirming this association holds significant importance for advancing women’s health and reducing cervical cancer incidence.

Vulvovaginal candidiasis affects 70–75% of women in the reproductive age range, with 40–50% experiencing recurrences and 5–15% recurring instances (Cassone, 2015). *Candida* spp., a commensal yeast in the human microbiota, becomes pathogenic under specific conditions (Lott et al., 2003). Elevated progesterone increases vaginal mucosa glycogen, serving as an energy source for yeast growth. Additionally, this phenomenon enhances the fungi’s adhesion capability (Sobel, 1993; Almeida Filho et al., 1995). Another study discovered a 59.5% incidence of C. albicans in HR-HPV-positive patients, below global (65.3%) and European (67.9%) prevalences (Pfaller et al., 2010). Previous studies found that *Candida* infection has been detected in around 25% of individuals diagnosed with HPV infection (Kinghorn, 1978; Voog et al., 1995). *Candida* (19.58%) was isolated at low frequencies in our study, consistent with global literature (Kinghorn, 1978; Voog et al., 1995; Pfaller et al., 2010). While the association between HPV and vaginal candidiasis remains controversial, some studies have suggested a potential link. A 213-case study showed vaginal candidiasis positively linked to HPV in South African young females (Onywera et al., 2022). However, a 4,449-woman study found no HPV-vulvovaginal candidiasis association (Wang et al., 2020). Fungal community shifts associate with HPV infections. Interplay between host, behaviors, *Candida* overgrowth, Lactobacillus requires more research to elucidate HPV pathogenesis mechanisms (Rosa et al., 2021).

In our study, we employed multinomial logistic regression analysis to evaluate the risks associated with HPV and *Candida* infections. The results revealed a substantial increase in the risks of LSIL, HSIL, and cervical cancer among individuals with HPV positivity, with fold increases of 7.45, 82.36, and 12.07, respectively. Conversely, those with *Candida* positivity showed a significant decrease in risks, with fold decreases of 0.75, 0.65, and 0.61. When comparing individuals without concurrent *Candida* and HPV infections, those solely afflicted with HPV exhibited significantly heightened risks for LSIL, HSIL, and cervical cancer, with fold increases of 7.6, 73.68, and 13.57, respectively. No significant difference was found between individuals without *Candida* and HPV infections and those solely afflicted with *Candida*. However, individuals co-infected with both HPV and *Candida* demonstrated significantly increased risks for LSIL, HSIL, and cervical cancer, with fold increases of 5.61, 47.88, and 7.8. *Candida* is an opportunistic pathogen that typically does not cause disease on the vaginal mucosa but may do so when the body’s immunity is compromised or the vaginal acidic environment is altered (Pfaller et al., 2010). Whether the natural history of HPV infection is affected by VVC infection has not been fully investigated. In one meta-analysis, VVC is a protective factor for HPV infection and had no correlation with CIN (Song et al., 2007). However, in this meta-analysis, only two studies including 444 patients with VVC infection were analyzed to explore the association between VVC and CIN (Caiyan et al., 2012; Rakislova et al., 2020). For cervical cancer, the multiplicative scale indicates a 0.37-fold decrease in risk, but the confidence interval was wide. These findings suggest a negative interaction, indicating that the combined effect of HPV and Fungi co-infection is less than the sum of their individual effects on cervical lesion development.

To delve deeper into the underlying molecular mechanisms, we investigated the combined impact of co-infections on the expression of p16 and Ki67 in the cervical region. P16 is consistently upregulated in high-grade squamous intraepithelial lesions (HSIL) and carcinomas driven by high-risk human papillomavirus (HR-HPV), triggered by HPV E6 and E7 proteins (Leeman et al., 2020; Nicolás et al., 2020; Rakislova et al., 2020). These proteins deactivate the retinoblastoma protein and p53, promoting cell proliferation and triggering a compensatory increase in p16 expression (Leeman et al., 2020; Singh and Gilks, 2020). Research has shown that *Candida albicans* can co-infect with HPV, affecting P16 protein expression—a hallmark of HPV infection. Co-infection significantly decreased P16 expression, suggesting *Candida albicans* potentially inhibits HPV replication/expression. Although the precise molecular mechanism is unclear, understanding how *Candida albicans* suppresses HPV could enable therapeutic developments. However, the combined infections did not show a significant impact on the expression of Ki67. Takkem et al. (2018) observed Ki67 differences with dysplasia degree. He et al. (2015) linked Ki67 to stage, grade, and cervical lymph node metastasis. Jing associated Ki67 with poorly differentiated tumors and cervical lymph node metastasis (Jing et al., 2018, 2019). However, in our study, no significant impact of *Candida* on Ki67 expression was observed. Speculatively, this lack of effect might be linked to an insufficient sample size. Further exploration through mechanistic studies and larger sample sizes is warranted.

Several limitations should be considered when interpreting the findings of this study. First, as a cross-sectional study, it is difficult to determine causal relationships between cervical fungal infections and lowered HPV persistence or cervical lesion incidence. Longitudinal cohort studies tracking these dynamics over time would better define protective or harmful fungal roles. Additionally, although ThinPrep Cytology Test (TCT) enables detecting cervical fungi missed by traditional vaginal swab analysis, culture-based quantitation of *Candida albicans* alongside genetic sequencing would provide invaluable specificity. Furthermore, while reduced P16 with stable Ki67 levels hints at HPV suppression, directly assaying HPV mRNA or protein levels would definitively confirm fungal effects on viral loads. Finally, as an observational clinical study, many unaccounted variables including patient sexual behaviors, microbiome composition, and immune health could distort perceived fungal interactions with HPV. Ultimately, uncovering *Candida albicans*’ ability to temper HPV disease warrants comprehensive investigations accounting for complex interactions between this fungus, virus, and the host immune system. Monitoring immunological fluctuations alongside HPV viral load changes following co-infection will undoubtedly provide pivotal pieces to solve this puzzle.

## 5 Conclusion

In conclusion, this study revealed a potential protective role of co-infection with fungi and HPV against cervical lesions compared to HPV infection alone in our study population. However, further research is needed to determine if these findings can be generalized to broader populations. The observed risk reduction for low-grade squamous intraepithelial lesions (LSIL), high-grade squamous intraepithelial lesions (HSIL), and cervical cancer underscores the impact of fungal co-infection. Interaction analysis suggested a negative interaction, indicating a sub-additive effect where fungal infection might mitigate HPV’s promoting effect on cervical lesions. Furthermore, lower p16 positivity in the co-infection group hints at a potential mechanism for this protective effect. These findings highlight a novel clinical interaction between cervical fungi and oncogenic HPV, prompting further investigation into their underlying mechanisms and therapeutic implications.

## Data Availability

The raw data supporting the conclusions of this article will be made available by the authors, without undue reservation.

## Footnotes

1
http://www.R-project.org
